# Future Prospects in the Treatment of Parasitic Diseases: 2-Amino-1,3,4-Thiadiazoles in Leishmaniasis

**DOI:** 10.3390/molecules24081557

**Published:** 2019-04-19

**Authors:** Georgeta Serban

**Affiliations:** Pharmaceutical Chemistry Department, Faculty of Medicine and Pharmacy, University of Oradea, 29 Nicolae Jiga, 410028 Oradea, Romania; getaserban_2000@yahoo.com; Tel.: +4-0756-276-377

**Keywords:** 2-amino-1,3,4-thiadiazole, neglected tropical diseases, protozoan parasites, *Leishmania* spp., antileishmanial activity, inhibitory concentration

## Abstract

Neglected tropical diseases affect the lives of a billion people worldwide. Among them, the parasitic infections caused by protozoan parasites of the *Trypanosomatidae* family have a huge impact on human health. Leishmaniasis, caused by *Leishmania* spp., is an endemic parasitic disease in over 88 countries and is closely associated with poverty. Although significant advances have been made in the treatment of leishmaniasis over the last decade, currently available chemotherapy is far from satisfactory. The lack of an approved vaccine, effective medication and significant drug resistance worldwide had led to considerable interest in discovering new, inexpensive, efficient and safe antileishmanial agents. 1,3,4-Thiadiazole rings are found in biologically active natural products and medicinally important synthetic compounds. The thiadiazole ring exhibits several specific properties: it is a bioisostere of pyrimidine or benzene rings with prevalence in biologically active compounds; the sulfur atom increases lipophilicity and combined with the mesoionic character of thiadiazoles imparts good oral absorption and good cell permeability, resulting in good bioavailability. This review presents synthetic 2-amino-1,3,4-thiadiazole derivatives with antileishmanial activity. Many reported derivatives can be considered as lead compounds for the synthesis of future agents as an alternative to the treatment of leishmaniasis.

## 1. The Significance of Thiadiazole Derivatives for Medicinal Chemistry

### 1.1. Biological Significance of Heterocyclic Compounds

Heterocyclic compounds are very common in biology. A large number of naturally occuring substances which are essential for living cells belong to the family of heterocycles. Some of them, such as amino acids, proteins, pyrimidine and purine bases of DNA, enzyme co-factors, oxygen-carrying pigment haemoglobin, photosynthesizing pigment chlorophyll, etc play a vital role in the metabolism of all living organisms and are essential for almost every stage of the biochemical processes necessary to support life [1,2]. Their biological activity is partly due to the wide range of their interactions, possibly through their heteroatoms which can act as acids or bases depending on the pH of the environment. In addition, the ability of heterocycles to engage in a wide variety of intermolecular interactions such as hydrogen bonds, metallic coordination bonds, van der Waals and hydrophobic forces, as well as their wide range of ring sizes, allow them to follow different patterns to enzyme binding and to fit into the various enzyme binding pockets structures, making them important scaffolds for drug development [2].

### 1.2. Thiadiazole Derivatives with Medical Significance

**Figure 1:**
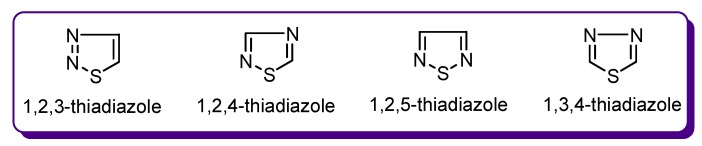
The isomers of the thiadiazole ring.

Nitrogen-containing heterocycles are widely distributed in Nature [3,4,5,6]. Many of these are present in plants and are known as alkaloids, some of which have been used since ancient times as medicinal agents. Thiadiazole derivatives belong to an important family of compounds having a common nitrogen-heterocyclic scaffold which is incorporated into the core structure of several natural products and medicinally significant synthetic compounds [7]. Thiadiazole is a five membered ring system containing sulphur and nitrogen atoms and occurs in four isomeric forms (e.g., 1,2,3-thiadiazole, 1,2,4-thiadiazole, 1,2,5-thiadiazole and 1,3,4-thiadiazole) (Figure 1).

**Figure 2:**
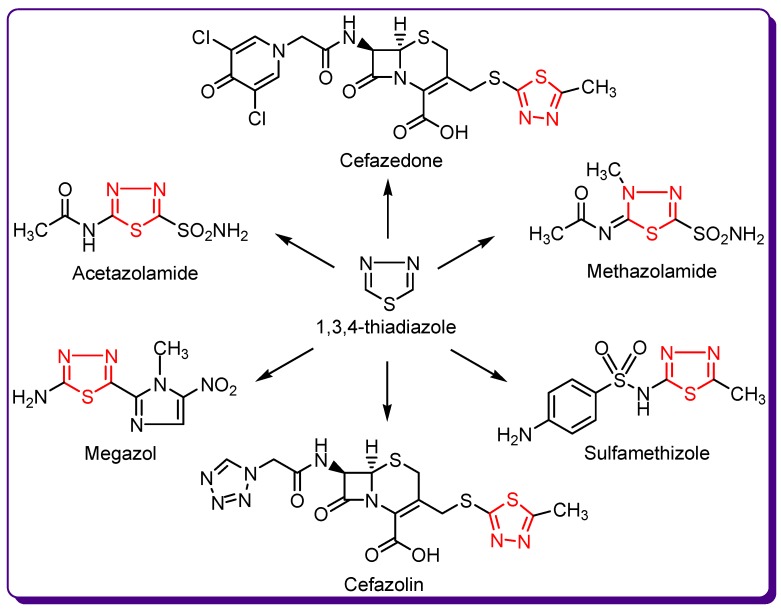
Drugs containing a 1,3,4-thiadiazole ring.

There are currently some marketed pharmaceutical products containing thiadiazole derivatives. Acetazolamide and methazolamide are potent carbonic anhydrase inhibitors, megazol is an antitrypanosomal agent and sulfamethizole possess antimicrobial activity. Cefazolin and cefazedone belong to the first generation of the cephalosporin family (Figure 2) [7,8,9].

**Figure 3:**
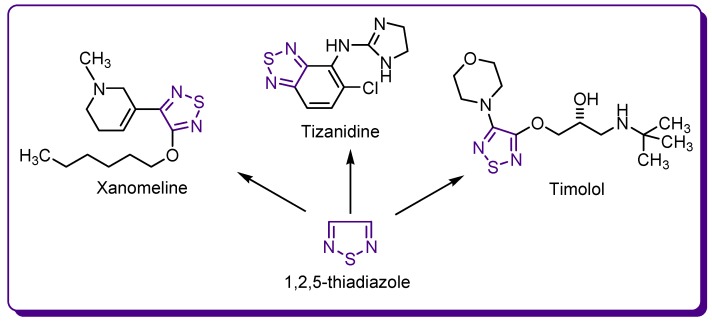
Drugs containing a 1,2,5-thiadiazole ring.

Timolol is a non-selective β-adrenergic blocking agent frequently used in the treatment of ocular hypertension and glaucoma. Tizanidine is an agonist of α2-adrenergic receptors, which decreases the release of excitatory adrenergic neurotransmitters from spinal cord interneurons and reduces the muscle spasticity in multiple sclerosis or spinal cord injury [8,10]. Xanomeline is a selective agonist of M1 and M4 acetylcholine receptor subtypes with antidopaminergic effects and antipsychotic-like profile, effective in schizophrenia and Alzheimer’s type dementia (Figure 3) [11,12,13].

**Figure 4:**
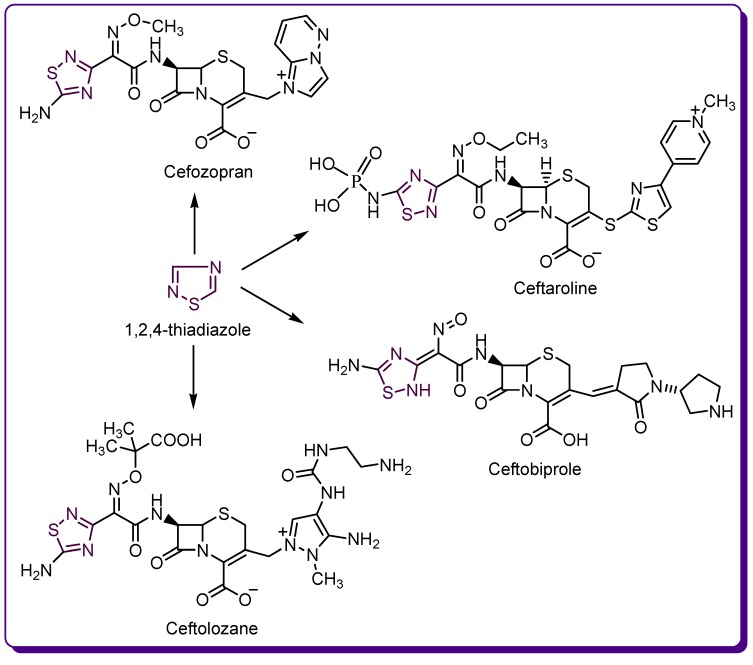
Drugs containing a 1,2,4-thiadiazole ring.

Despite the numerous studies on the biological potential of 1,2,4-thiadiazole derivatives [14,15], the only drugs containing the 1,2,4-thiadiazole ring belong to the last generations of cephalosporin antibiotics, such as cefozopran—a fourth-generation cephalosporin and ceftaroline, ceftobiprole and ceftolozane as examples of the fifth generation cephalosporins (Figure 4) [16,17,18,19].

**Figure 5:**
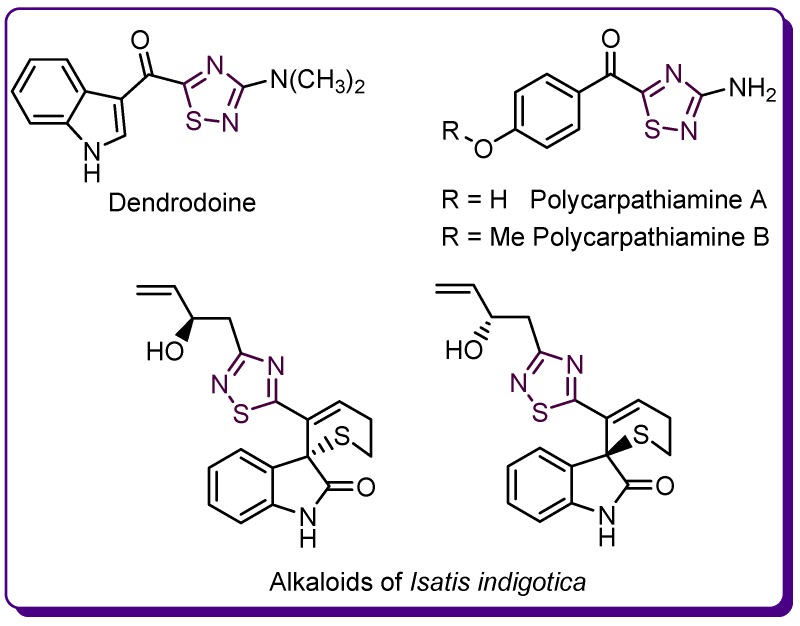
Natural thiadiazole derivatives.

Among the thiadiazole isomers, 1,2,4-thiadiazole is the only one found in the known natural compounds. Therefore, dendrodoine—an indole alkaloid isolated from the marine tunicate *Dendrodoa grossularia* (*Styelidae* family), has been the only known 1,2,4-thiadiazole derivative for over thirty years [20,21,22]. Recently, two new alkaloids featuring the 3-amino-5-acyl-1,2,4-thiadiazole moiety, polycarpathiamine A and B, were isolated from the ascidian *Polycarpa aurata (Styelidae* family) [22]. In addition, a pair of enantiomers of an indole alkaloid containing dihydrothiopyran and 1,2,4-thiadiazole rings were isolated from the root extract of *Isatis indigotica* (*Cruciferae* family) (Figure 5) [23].

Thiadiazole is a versatile moiety and the thiadiazole derivatives have been widely studied for medical, agricultural and industrial applications such as bioactive compounds, herbicides, metal chelating agents, corrosion inhibitors, cross-linkers for polymers, etc. [1,24,25,26]. The thiadiazole moiety acts as ”hydrogen binding domain” and ”two-electron donor system”. It also acts as a bioisostere of pyrimidine, pyridazine, oxadiazole, oxazole, thiazole and benzene, and this property can increase the biological potential and lead to analogues with higher lipophilicity due to the sulfur atom which imparts improved liposolubility [9,25,26,27,28]. Among the four isomers of the thiadiazole ring, 1,3,4-thiadiazole derivatives are the most used in therapy and are most studied because of the broad spectrum of pharmacological activities, probably due to the =N–C–S– group [9,29]. Moreover, the mesoionic nature of 1,3,4-thiadiazoles which allows them to behave as masked dipoles, enables these compounds to have strong interactions with biomolecules (e.g., DNA, proteins). Despite of their internal charges, the mesoionic compounds are neutral and are able to cross cellular membranes, this fact also contributing to the good cell permeability of 1,3,4-thiadiazole derivatives [9,25,30].

### 1.3. Studies on the 2-Amino-1,3,4-thiadiazole Series

2-Amino-1,3,4-thiadiazole derivatives have been previously studied for potential anticancer activity [31,32,33,34,35] and some of them were introduced in clinical trials for the treatment of patients with different types of cancer [7,36,37,38,39]. Although the clinical studies have shown weak activity compared to the preliminary observations, the 2-amino-1,3,4-thiadiazole moiety proved to be a good scaffold for future anticancer agents. In addition, it has been reported that several 2-amino-1,3,4-thiadiazole derivatives exhibit promising antimicrobial activity [9]. As a continuation of our preoccupation in the field of biologically active thiadiazoles [9,40,41,42,43,44,45], the purpose of this review is to highlight the antileishmanial activity exhibited by small molecules possessing a 2-amino-1,3,4-thiadiazole moiety. This review also provides information on structure-activity relationship (SAR) studies and the mechanisms of action of some derivatives.

## 2. Kinetoplastea Parasites

### 2.1. Kinetoplastid Infections

A number of protozoan pathogens are human parasites and represent a significant threat to health causing more than one million deaths annually. They also threaten the lives of billions of people worldwide and are associated with significant morbidity and major economic impact [46,47]. According to the World Health Organization (WHO), neglected tropical diseases affect the lives of a billion people worldwide [48]. Among them, the parasitic infections caused by trypanosomatids have a huge impact on human health. Kinetoplastea is a class of flagellated parasitic protozoans, which includes clinically important human pathogens that are vectored by insects [49]. The three major human diseases resulting from kinetoplastid infections include human African trypanosomiasis (sleeping sickness) which is caused by *Trypanosoma brucei gambiense* and *Trypanosoma brucei rhodesiense*, American trypanosomiasis (Chagas disease) caused by *Trypanosoma cruzi*, and various forms of leishmaniasis caused by different species of *Leishmania* [50]. These three neglected tropical diseases account for over 60,000 human deaths per year and involve about 4 million disability-adjusted life years (DALYs) [47,48,49,51]. There are currently no vaccines against leishmaniasis and other trypanosomatid diseases. The treatment and prophylaxis depend on a limited range of drugs and many of which have become less effective due to increasing drug resistance [47,52,53]. These protozoan diseases are among the most neglected parasitic diseases in terms of drug discovery and development and the number of people affected by them [51,52].

### 2.2. Leishmania Parasites and Leishmaniasis

The leishmaniases are a group of tropical diseases caused by protozoan parasites of the genus *Leishmania* in the *Trypanosomatidae* family [54]. There are more than 20 Leishmania species that are transmitted to humans through the bite of infected female sandflies of the *Phlebotomus* genus (in the Old World) or *Lutzomyia* (in the New World) [55,56]. *Leishmania* parasites have a life cycle that includes an extracellular flagellated promastigote stage and an obligatory intracellular, non-motile amastigote stage. The elongated promastigotes propagate in the insect gut and are transmitted to mammalian hosts through the bite of sandflies. In mammalian hosts, the parasites are rapidly transformed into round non-flagellated amastigotes within mononuclear phagocytes [57,58].

Leishmaniasis is endemic in over 88 countries on four continents and is closely associated with poverty [58]. There are different forms of the disease, such as visceral leishmaniasis (VL—also known as kala-azar), post-kala-azar dermal leishmaniasis (PKDL), cutaneous leishmaniasis (CL) and mucocutaneous leishmaniasis (MCL). Cutaneous leishmaniasis is the most common form, while the visceral leishmaniasis is the most severe form and can be fatal if untreated [56,59]. About 0.7–1.2 million new cases of cutaneous leishmaniasis and 50,000–90,000 new cases of visceral leishmaniasis are reported every year [59,60]. Leishmaniases remain as major diseases in much of tropical and subtropical countries, where 310 million people are at risk and 12 million people are infected [53,60].

*Leishmania amazonensis* has been associated to all clinical forms of leishmaniasis. *Leishmania major, Leishmania tropica, Leishmania aethiopica* and *Leishmania mexicana* are associated with cutaneous leishmaniasis. Mucocutaneous leishmaniasis is caused by *Leishmania braziliensis*, while the visceral leishmaniasis is caused by three related species or subspecies: *Leishmania donovani*, *Leishmania donovani infantum* and *Leishmania donovani chagasi* which form the *Leishmania donovani* complex [26,61]. These protozoan parasites produce a systemic and life-threatening infection by infecting reticuloendothelial cells and macrophages in all organs. The infection may be subclinical, but clinical disease is fatal if untreated [57]. There is a recent increase in leishmaniasis spreading due to the high cost of drugs and coinfection with human immunodeficiency virus (HIV) [53,57].

### 2.3. Current Pharmacotherapy for Leishmaniasis

The classical treatment of leishmaniasis is limited to toxic, high price or poorly tolerated drugs, such as pentavalent antimonial derivatives (sodium stibogluconate—Pentostam, *N*-methyl- glucamine antimoniate—Glucantime), pentamidine and amphotericin B. Liposomal amphotericin B, paromomycin and miltefosine are active drugs against leishmaniasis that have arisen via repurposing [47]. Edelfosine, ilmofosine, sitamaquine, and some plants products also proved clinically important antileishmanial activity [54].

The pentavalent antimonial derivatives are antimony-carbohydrate complexes and have been used for all clinical forms of leishmaniasis for more than 70 years [62,63]. The mechanism of action of pentavalent antimonials has remained essentially unknown. Evidence suggests that pentavalent antimony Sb(V) is a prodrug which is reduced in vivo into trivalent antimony Sb(III) [63,64]. Sb(III) is the active/toxic form that exhibits antileishmanial activity. This reduction can occur in macrophages as well as in *Leishmania* parasites. It seems that amastigotes can reduce Sb(V), while promastigotes cannot, making amastigotes more sensitive to Sb(V) [65]. The reduction of Sb(V) to Sb(III) is favored by slightly elevated temperature and acidic pH. This suggests that Sb(V) drugs may act as a molecular carrier releasing the active Sb(III) form in the acidic intracellular compartments where the *Leishmania* parasites reside [64,65]. The antileishmanial activity of Sb(III) involves multiple pathways, such as: interaction with thiol metabolism of parasites resulting in deoxyribonucleic acid (DNA) fragmentation; inhibition of *Leishmania* type I DNA topoisomerase which promote adenosine triphosphate (ATP) and guanosine triphosphate (GTP) depletion resulting in the reduction or depletion of parasites energy; promotion the production of proinflammatory cytokines that can increase the release of reactive oxygen species [63].

Even though pentavalent antimonial drugs are still the first-line medication in several developing countries, they have several limitations due to the toxicity of Sb(III) [64]. These drugs require daily parenteral administration which causes local pain. Systemic side effects such as hepatotoxicity, nephrotoxicity, pancreatitis, cardiac toxicity, myalgia, abdominal colic, nausea, vomiting, diarrhea are common during treatment [63,64]. In addition, the development of drug resistance decreases the effectiveness of these compounds [63,64,65]. The use of pentamidine is limited due to toxicity and drug resistance [47].

Conventional amphotericin B has replaced antimonial drugs and is highly effective against antimonial-resistant VL, although it is toxic and requires slow parenteral administration [47,62,63]. Amphotericin B is an antimycotic polyene macrolide that binds to the ergosterol in the cell membrane leading to parasite membrane disruption [62] and alteration of membrane permeability, followed by cell death [65]. Due to its poor aqueous solubility, low acid stability and low intestinal absorption, its use is restricted to slow intravenous infusion [62,63,65]. Its mechanism of action is also responsible for its toxicity. Amphotericin B can form complexes with membrane sterols (e.g., ergosterol in *Leishmania* cell membrane and cholesterol in mammalian cell membrane) resulting in cell damage [63]. Therefore, amphotericin B can cause serious side effects including nephrotoxicity, thrombophlebitis and myocarditis [47,65]. Liposomal amphotericin B was approved in 1997 [47,63]. Incorporation of amphotericin B into lipidic systems has improved its antimicrobial efficacy and reduced its toxicity. The liposomal amphotericin B accumulates in macrophages and therefore become a good treatment for leishmaniasis. However, the high cost of this drug is a serious limitation in many regions [47,62,63].

Paromomycin is an aminoglycoside antibiotic with broad-spectrum against Gram-positive and Gram-negative bacteria. The antileishmanial activity of paromomycin was discovered in the 1960s [47]. Paromomycin binds to the 30S ribosomal subunit, resulting in inhibition of protein synthesis [47,62]. The mechanism of antileishmanial activity of paromomycin is not very clear. Some studies suggest that cationic paromomycin binds to the negatively charged leishmanial glycocalyx and lipophosphoglycan, a major component of the *Leishmania* promastigotic cell surface [65]. Due to low oral bioavailability and rapid clearance, paromomycin requires frequent parenteral administration causing nephrotoxicity, ototoxicity and hepatotoxicity [65]. In addition, the susceptibility of *Leishmania* spp. to paromomycin is geographically variable [62]. Therefore, paromomycin remains an option for the treatment of leishmaniasis [47].

Miltefosine, the phosphorylcholine ester of hexadecanol, is the only drug available for oral treatment of leishmaniasis [47,65]. Miltefosine was originally developed as an anticancer agent for local treatment of cutaneous metastases of breast cancer and other types of tumors. Its study was discontinued due to dose-limiting gastrointestinal side effects [47]. However, miltefosine was reinvestigated and is the most recent antileishmanial drug. Although its mechanism of action is not clearly understood, there is some evidence that it targets parasitic enzymes such as mitochondrial cytochrome C oxydase and glycosomal alkyl-specific acyl-CoA acyltransferase. Miltefosine causes disturbances in ether-lipid metabolism, glycosylphosphatidylinositol anchor biosynthesis and signal transduction in *Leishmania* [63]. Oral administration is a particularly attractive quality of miltefosine [63]. However, its use might be limited by high cost and adverse effects such as gastrointestinal toxicity, nephrotoxicity, increased levels of hepatic enzymes and teratogenity [47,62,63].

New potent, low-cost and orally available medicines are needed to improve the condition of patients and to control the parasites responsible for parasitic diseases. The development of antileishmanial drugs has been accelerated in recent years through the establishment of the World Health Organization’s Program for Neglected Tropical Diseases [47].

## 3. Antileishmanial Activity Associated with 2-Amino-1,3,4-thiadiazole System

### 3.1. The Significance of Hydroxyl, Oxyalkyl and Aminoalkyl Side Chains for Antileishmanial Activity

The antileishmanial properties of amino-1,3,4-thiadiazole derivatives have been reported and several mechanisms of action have been postulated [57,60,66,67,68]. Thus, Al-Qahtani et al. [57] have reported several analogs such as **1**–**3**, having a 1,4-dihydroxyphenyl or 1,4-dimethoxyphenyl group attached to the 1,3,4-thiadiazole ring. The proliferation screening of *L. donovani* promastigotes has shown that the compounds have a very promising in vitro antileishmanial effect, even at concentrations as low as 50 μM. It has been observed that the activity increased at higher concentrations (100, 200 and 400 μM), when all the tested compounds exhibited an inhibitory potency comparable to that of amphotericin B. The microscopic observation revealed that the promastigotes were non-motile when were incubated with the test compounds. 2-[(5-Phenyl- amino)-1,3,4-thiadiazol-2-yl]benzene-1,4-diol (**1**) was the most active derivative at each concentration. The authors suggested that the 1,4-dihydroxyphenyl and 1,4-dimethoxyphenyl groups might be involved in the mechanism of action of these derivatives. Due to the ability of these groups to form free radicals, these compounds could permeate the cell membrane and damage the nucleic acids, proteins or other important molecules for the parasites survival. In addition, the affinity of 1,3,4-thiadiazole derivatives for the sulphydryl groups of enzymes or proteins is known. Thus, the inactivation of parasitic enzymes or proteins that have cysteine residues following the thiadiazole attack may be another mechanism of action that deserves attention [57].

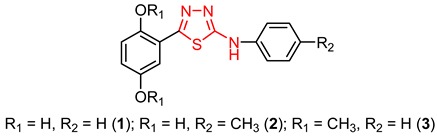



The significance of hydroxyl and methoxy groups for the antileishmanial activity of some synthetic compounds can be also suggested from the studies made by Tahghighi et al. [52,60]. Among the 5-(5-nitrofuran-2-yl)-1,3,4-thiadiazol-2-amines **4**–**15** and 5-(5-nitrothiophen-2-yl)-1,3,4- thiadiazol-2-amines **16**–**25**, the most active derivatives possess a hydroxypropyl and a methoxypropyl moiety, respectively, in the side chain (derivatives **19** and **20**—the half maximal inhibitory concentration IC_50_ values of 3 μM compared to the reference drugs, glucantime—IC_50_ value of 68.44 mM and fluconazole—IC_50_ value of 941.1 μM against promastigote form of *L. major*) (Table 1) [52].

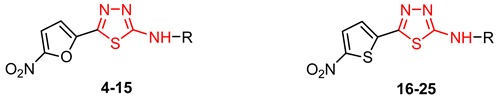



The activity against amastigote form of *L. major* has also been investigated (in murine peritoneal macrophage) for compounds showing a good activity against promastigotes (IC_50_ values < 45 μM) and all tested compounds have shown significant activity. The most active derivatives against promastigotes, **19** and **20**, were also the most effective compounds against amastigotes. The derivatives **19** and **20** significantly reduced the number of intracellular amastigotes per macrophage (the mean number of amastigotes/macrophage after treatment with derivative **19** or **20** for 24 h ≈ 0.6–0.7), the percentage of macrophage infectivity (≈35–40%) and infectivity index (infectivity index of macrophages cultured for 24 h in the presence of derivatives **19** and **20**, respectively < 30) compared to the control group (the mean number of amastigotes/macrophage ≈ 1.7; the percentage of infected macrophages ≈ 75%; the infectivity index of macrophages ≈ 130). In addition, the compound **7** (IC_50_ value of 21 μM) has also been ranked among the most effective derivatives against amastigotes in terms of the percentage of macrophage infectivity (≈35%) and infectivity index (infectivity index < 30) [52].

In vitro antileishmanial experiments against both promastigote and amastigote forms of *L. major*, followed by structure-activity relationship studies, revealed that an acyclic amine at C-2 position of 1,3,4-thiadiazole ring connected to a distal hydrophilic atom (O or N) in the side chain has major role in the biological activity. Hence, compounds bearing a simple alkyl side chain (e.g., methyl, ethyl, *c*-propyl) were less potent compared to oxyalkyl and aminoalkyl derivatives. SAR studies showed that potent compounds possess an aliphatic chain of 2–4 carbons between two heteroatoms (nitrogen-oxygen and nitrogen-nitrogen, respectively) in the side chain. It can be concluded that the insertion of oxygen or nitrogen into the alkyl side chain improves antileishmanial activity [52].

### 3.2. The Significance of a Supplementary Heterocycle for the Antileishmanial Activity

The studies made by Tahghighi et al. [52,60] also offered some information about the significance of the heterocycle linked to the C-5 position of the 1,3,4-thiadiazole ring. The 5-nitrofurans **4**, **5** and **6** bearing small alkyl side chains exhibited some antileishmanial activity (IC_50_ values of 50–55 μM), while the corresponding 5-nitrothiophene derivatives **16**, **17** and **18** were less active (IC_50_ values of 64–98 μM). Concerning the oxyalkyl and aminoalkyl derivatives, the results were generally better for the 5-nitrofuran derivatives **4**–**15** (e.g., compounds **7**–**13** and **15**). These studies confirmed that better antiinfective properties are exhibited by the compounds having a 5-nitrofuryl moiety, even though there are some exceptions. Thus, the most active derivatives **19** and **20** belong to the 5-nitrothiophene series. The hydroxypropyl derivative **19** was six times more potent than its 5-nitrofuran analog **8**, while the derivative **24** was three times more potent than its 5-nitrofuran analog **14**.

In vitro cytotoxicity determined against the mouse peritoneal macrophages for compounds exhibiting good antipromastigote activity (IC_50_ values < 45 μM) showed macrophage toxicity for many derivatives (cytotoxic concentration for 50% inhibition CC_50_ values < 100 μM). There are derivatives, such as **7**, **11**, **15** and **25** which showed a low level of toxicity (CC_50_ values > 100 μM) or even tolerable toxicity to mouse peritoneal macrophages (derivatives **10**, **13** and **23**, CC_50_ values > 80 μM). The most active derivatives **19** and **20** showed toxicity against macrophage. However, these compounds showed the highest selectivity index (SI = CC_50_/IC_50_, SI values of 14.05 and 12.60, respectively). Interesting results were obtained for compound **15** which showed the lowest cytotoxicity (IC_50_ value of 33 μM, CC_50_ value of 367.65 μM and SI value of 11.14) (Table 1) [52].

As the biological studies have shown, the antileishmanial activity is also maintained when the distal nitrogen is included in heteroaromatic rings such as 1,2,3-triazole or pyridine [52,69]. In the series of *N*-[(1-benzyl-1*H*-1,2,3-triazol-4-yl)methyl]-5-(5-nitrofuran-2-yl)-1,3,4-thiadiazol-2-amines **26**–**34** which were evaluated for their in vitro antileishmanial activity against promastigote (extracellular parasite) and amastigote (intramacrophage parasite) forms of *L. major*, 4-methylbenzyl analog **32** was the most potent compound (IC_50_ value of 12.2 μM) [69].

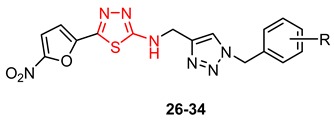



C-2 amine in these compounds is also acyclic, but 1,2,3-triazole ring acts as a nitrogen- containing heterocycle in the distal position. The unsubstituted benzyl derivative **26** showed good activity (IC_50_ value of 19.6 μM) and introduction of different substituents on the benzyl group slightly increased or even decreased the biological activity. Significant activity (IC_50_ values < 20 μM) was exhibited by halogenated derivatives having 2-halo-substitutions (e.g., derivatives **27**, **29**, **33**, **34**) (Table 2) [69].

The compounds exhibiting a good antipromastigote activity (IC_50_ values < 25 μM) were also evaluated for their activity against amastigote form of *L. major* in murine peritoneal macrophage. All tested compounds showed a significant antiamastigote activity. Compound **32**, the most active derivative against promastigotes, was also the most effective compound against amastigotes. The derivative **32** significantly decreased the number of intracellular amastigotes per macrophage (the mean number of amastigotes/macrophage after treatment with derivative **32** for 24 h ≈ 0.7), the percentage of macrophage infectivity (≈33%) and infectivity index (≈25) compared to the control group (the mean number of amastigotes/macrophage ≈ 2.1; the percentage of infected macrophages ≈ 77%; the infectivity index of macrophages ≈ 160) [69].

The replacement of the 1,2,3-triazole ring in the side chain with pyridine was well-tolerated. The in vitro antileishmanial studies showed good activity for pyridine-3-yl derivative **35** (IC_50_ value of 26 μM, CC_50_ value of 41.72 μM) and pyridine-2-yl derivative **36** (IC_50_ value of 36 μM, CC_50_ value of 111.71 μM) [52]. These results demonstrate that C-2 acyclic amine substituents have a high flexibility for chemical modifications and they determine the biological potency of the derivatives. The flexibility and conformational adaptation provided by this side chain can be another explanation for the biological activity of these compounds. By adding suitable substituents to the C-2 acyclic amine of 1,3,4-thiadiazole ring, the pharmacological and physicochemical properties of these compounds could be optimized [52,60,69].

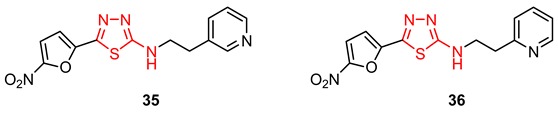



### 3.3. Mesoionic Derivatives of 2-amino-1,3,4-thiadiazole Moiety

Mesoionic compounds are a special class of heterocycles that have provided many derivatives with a wide range of biological activities, including anti-inflammatory, analgesic, antibacterial, antifungal or antitumor activities [67]. Some mesoionic derivatives of the 1,3,4-thiadiazolium-2-aminide class such as **37**–**45** inhibited the in vitro growth of promastigote and amastigote forms of *L. amazonensis* [67]. All the compounds were more effective against the extracellular stage of the parasites (promastigote) compared to intracellular stage (amastigote). The authors found a few compounds (e.g., derivatives **37**, **43** and **44**—IC_50_ values of 0.47–0.52 μM) as active as the reference drug, pentamidine (IC_50_ value of 0.46 μM). Among the studied compounds, the most active against promastigote forms were the alkoxy derivatives 4-methoxy **38** and 3-methoxy **42** with IC_50_ values of 0.17 μM and 0.04 μM, respectively. Concerning the amastigote forms, the most active compounds were the halogenated derivatives 4-fluor **39** and 3-bromo **44** with IC_50_ values of 5.37 μM and 5.48 μM, respectively compared to pentamidine (IC_50_ value of 118 μM) (Table 3) [67].

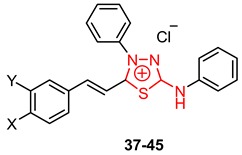



A major difference in the sensitivity of both life stages of *L. amazonensis* was observed for all tested compounds. The high sensitivity of promastigotes compared to amastigotes can suggest the different mechanisms in the antileishmanial activity. It seems that inhibition depends on the differences between the promastigotes and amastigotes surface structure. In addition, the electronic and lipophilic parameters of the derivatives as well as the pH value of the experimental medium can influence the biological activity [67].

Furthermore, the effectiveness of mesoionic derivatives **37**–**45** against *L. braziliensis* and *L. chagasi* was investigated. The general activity profile of the compounds showed that the biological activity was influenced by the type of the group attached to the 3- and 4-position of the 5-cinnamoyl-1,3,4-thiadiazole nucleus. Particular differences in their median effective dose (ED_50_) values were observed. Thus, the derivatives bearing electron-donating groups such as methoxy (derivatives **38** and **42**) exhibited very potent activity against one species (*L. amazonensis*), while the derivatives bearing electron-withdrawing groups such as halogen (derivatives **39**, **40**, **41**, **43**) exhibited very potent or potent activity among the three species (*L. amazonensis*, *L. braziliensis* and *L. chagasi*) compared to pentamidine (ED_50_ value of 23.64 μM for *L. amazonensis*, 64.18 μM for *L. braziliensis* and 27.5 μM for *L. chagasi*).

The idea of developing a single effective drug against all forms of leishmaniasis seems unfeasible. Among the halogens, the fluorine is the strongest electron-withdrawing element and the derivative **39** was the most active compound against two species of *Leishmania* (IC_50_ values of 5.37 μM against *L. amazonensis* amastigotes and ED_50_ value of 3.42 μM against *L. chagasi*, respectively). The cytotoxic effect of these compounds was investigated on mouse peritoneal macrophages and expressed by the median toxic dose (TD_50_), showing greater safety compared to pentamidine for many of the studied derivatives. The compounds **39**, **40**, **41** and **43** exhibited high in vitro leishmanicidal activity combined with lower toxicity and therefore these compounds could be promising scaffolds to develop new therapeutic strategies for leishmaniasis treatment (Table 3) [61].

Additionally, in vivo studies made in the mouse *L. amazonensis* cutaneous infection model showed that the 4-methoxy derivative **38** exhibited comparable activity to the reference drug, meglumine antimoniate [70]. The derivative **38** was more effective than the non-substituted derivative **37**. Thus, mice infected with *L. amazonensis* promastigotes (1.2 × 10^6^) were treated with derivative **37** (24 mg/kg/day), derivative **38** (22 mg/kg/day) and meglumine antimoniate (100 mg/kg/day with 28 mg pentavalent antimonial), respectively, by a subcutaneous route. Treatment started in the fourth week after infection and continued for four weeks. Progression of the lesions was monitored until the week 12th post infection by measurement of footpad swelling. At the end of drug administration (week eight), there was a slight difference between the groups of mice treated with test compounds (lesion size ≈ 2 mm) or meglumine antimoniate (lesion size ≈ 2 mm) and untreated infected mice (lesion size ≈ 3.5 mm). At the 10th week post infection, there was an increased difference in footpad swelling between the mice treated with the test compound **38** (derivative **38**, lesion size ≈ 3 mm; derivative **37**, lesion size ≈ 6 mm) or meglumine antimoniate (lesion size ≈ 4 mm) and untreated infected mice (lesion size ≈ 7 mm), respectively. At the 12th week post infection, the results showed significantly differences between the groups of mice treated with test compounds (derivative **38**, lesion size ≈ 6 mm; derivative **37**, lesion size ≈ 9 mm) or meglumine antimoniate (lesion size ≈ 7 mm) and untreated infected mice (lesion size ≈ 14 mm), respectively [70]. The authors concluded that no significant differences in lesion size were observed in the groups treated with mesoionic compounds or meglumine antimoniate [70].

The animals were killed at the end of the experiment. The popliteal lymph nodes and spleens were removed, treated with mesoionic derivatives **37** and **38** and meglumine antimoniate, respectively, and the number of parasites was estimated by the limiting-dilution technique. The number of parasites in both lymph nodes and spleens of animals treated with mesoionic derivatives or meglumine antimoniate were significantly reduced compared to those of untreated control animals. In both cases, the reduction in the number of parasites after treatment with mesoionic derivatives was greater than that observed after meglumine antimoniate treatment (i.e., the number of parasites/mg tissue of lymph node in untreated animals ≈ 13,000, the number of parasites/mg tissue of lymph node in animals treated with meglumine antimoniate ≈ 3000, the number of parasites/mg tissue of lymph node in animals treated with mesoionic derivatives **37** or **38** < 1000, respectively) [70]. This suggests that there is a control of infection progression and the dissemination is limited. No apparent hepatic or renal toxicity following the treatment with mesoionic compounds was observed as evidenced by normal level of aspartate aminotransferase (AST) (i.e., 51 for untreated mice, 53 for mice treated with derivative **38** and 48 for mice treated with derivative **37**), alanine aminotransferase (ALT) (i.e., 52 for untreated mice, 57 for mice treated with derivative **38** and 58 for mice treated with derivative **37**) and creatinine concentrations (2.81 for untreated mice, 2.56 for mice treated with derivative **38**, 2.84 for mice treated with derivative **37**) [70]. It is difficult to determine whether in vitro activity of a compound will correlate with its in vivo activity because many factors such as bioavailability, toxicity and drug metabolism may influence the animal model. Fortunately in this case, the potent in vitro activity of compound **38** was in good agreement with its in vivo activity against *L. amazonensis* [61].

### 3.4. 2-Amino-1,3,4-thiadiazole Derivatives as Parasite Enzyme Inhibitors

#### 3.4.1. Nitric Oxide Synthase Inhibitors

The healing of leishmaniasis appears to be dependent on the development of an effective immune response that activates macrophages to produce reactive nitrogen and oxygen metabolites to kill intracellular amastigotes. Imiquimod, which is an immunomodulator, proved to be a good adjunct to CL therapy [71]. Depending on their activation state, macrophages can either host or kill *Leishmania*. Inducible synthesis of nitric oxide (NO) and the presence of an oxidative burst are of crucial importance for the killing of parasites [71,72]. Experiments have revealed that NO-stimulated production by activated macrophages is the key factor in killing *Leishmania* intracellular parasites [71].

The chemistry of mesoionic rings, which allows them to behave as masked dipoles, has been an extensively researched area since the late 1950s. They possess a betaine-like character with a partial positive charge on the heterocyclic ring which is balanced by a negative charge located on an exocyclic atom. The large separation between the charged regions leads to large dipole moments. This structure with well-separated regions of positive and negative charge associated with a polyheteroatomic system, suggest a high probability of strong interaction with biomolecules such as DNA and proteins. Moreover, the overall neutral character of these compounds enables them to cross biological membranes [70,73,74]. It is known that some classes of mesoionic compounds such as sydnones, sydnonimines and oxatriazoles possess in vivo NO-releasing properties and their leishmanicidal mechanism of action seems to be via activation of macrophages. Several parasites are highly sensitive to NO and its derivatives. The thiadiazolium compounds are not NO donors but are capable to induce NO production in macrophage cultures resulting in killing or control of *Leishmania* parasites [61,74]. The function of NO in the leishmanicidal activity of activated macrophages has been demonstrated both in vitro and in vivo [75,76,77,78].

The experiments with the mesoionic derivatives showed a significant increase in NO production in lymph node cell culture supernatants of infected mice after treatment with derivative **38** (≈6 μM nitrite) compared to meglumine antimoniate (≈3.2 μM) and untreated mice (≈3.5 μM nitrite) [70]. NO production was assayed by measuring the nitrite concentrations present in the supernatant of the lymph node culture. These results might suggest that mesoionic compounds could activate the mechanisms that positively influence the host’s ability to remove the parasites from infected cells and to control the parasites dissemination [70]. On the other hand, there is evidence that *Leishmania* spp. can produce NO from L-arginine and the NO-*L. amazonensis* pathway is involved in the host-cell/parasite interaction. In vitro experiments to find intracellular targets of antileishmanial drugs revealed that mesoionic derivatives can decrease the NO production of the parasite. Thus, the methoxy derivatives **38** and **42**, which have been shown to be cytotoxic to *L. amazonensis*, reduced from 70 to 90% of the NO production of parasites and acted on a soluble nitric oxide synthase (NOS) purified from *L. amazonensis* promastigotes and axenic amastigotes. The reference drug, pentamidine isethionate, was able to inhibit only 2% of NO production [70,74].

The mesoionic 1,3,4-thiadiazolium derivatives **46**–**48**, tested on promastigotes and axenic amastigotes of *L. amazonensis*, were more effective against axenic amastigotes (X = H, OCH_3_ and NO_2_ and IC_50_ values of 9.5, 13 and 5.5 μM, respectively) compared to pentamidine (IC_50_ value of 118 μM). A low toxicity (~10%) to mouse peritoneal macrophage was observed compared to pentamidine (49%). In an attempt to define a potential molecular target, the activities of NOS and arginase of the parasites treated with the mesoionic derivatives were evaluated. Arginase hydrolyzes L-arginine to L-ornithine and urea and it favors parasite growth. L-Arginine plays a central role in the biosynthesis of various products. Thus, L-arginine is involved in the production of both NO, mediated by NOS, and L-ornithine, mediated by arginase. Since NO in micromolar concentration is cytotoxic for microbial organisms, it can be seen that NOS and arginase pathways have opposite biological effects. All derivatives were able to inhibit the parasite NOS, the nitro-derivative **48** being the most active (28% inhibition of the NADPH consumption by NOS from promastigotes and 41% inhibition of the NADPH consumption by NOS purified from axenic amastigotes). In comparison, pentamidine was able to inhibit about 15% of NADPH consumption by NOS purified from parasite. Arginase activity from both stages of the parasite was measured using urea production. None of the investigated compounds were able to inhibit the arginase activity of axenic amastigotes. When tested with promastigotes, only the unsubstituted derivative **46** inhibited arginase activity. Thus, the derivatives **46** and **48** may be used as prototype molecules for the development of new therapeutic agents with high efficacy and low cytotoxicity [79].

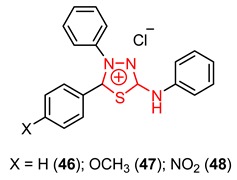



However, further studies to elucidate the mechanism of action of mesoionic compounds and to identify new targets for defending organisms against *Leishmania* infection are needed [66,74]. Indeed, many recent studies emphasized the significance of metabolic pathways that are both essential for parasite survival and absent within the host. These include the research focused on the importance of trypanothione pathway for the survival and infectivity of parasites [58,80,81].

#### 3.4.2. Inhibitors of Trypanothione Reductase/Tryparedoxin/Tryparedoxin Peroxidase System

**Figure 6:**
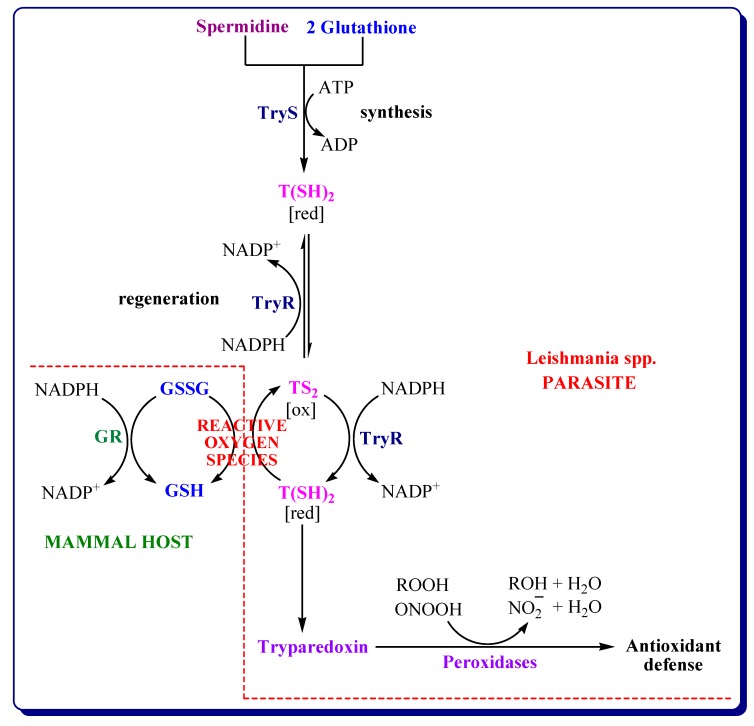
Glutathione and trypanothione-based redox systems in mammals and *Leishmania* spp. parasites. GSSG: glutathione disulfide, ROOH: alkyl-hydroperoxide, ONOOH: peroxynitrite, ROH: alcohol, NO_2_: nitrite (adapted from [81,82]).

Thiol-dependent redox metabolism is one of the unique metabolic features that distinguishes trypanosomatids from humans and provides reliable molecular targets for the selective development of drugs. An example of genetic features of trypanosomatids is the lack of genes encoding glutathione reductase (GR) and thioredoxin reductase, the main enzymes in the redox systems of most living organisms (i.e., the glutathione/glutaredoxin system and the thioredoxin system). Trypanosomatids are based, in turn, on trypanothione and flavoenzyme trypanothione reductase to support intracellular redox homeostasis [82]. Trypanothione (bis(glutathionyl)- spermidine, TS_2_—oxidized form of trypanothione, T(SH)_2_—reduced form of trypanothione) is an essential molecule for modulating oxidative stress in parasites. Trypanothione synthesis is catalyzed by two key enzymes: trypanothione synthetase (TryS) and trypanothione reductase (TryR). TryS is responsible for the synthesis of trypanothione from one molecule of spermidine and two molecules of glutathione using the energy provided by two ATP molecules. TryR maintains trypanothione in its reduced form T(SH)_2_ using NADPH as cofactor. NADPH can be supplied by the oxidative phase of the pentose phosphate pathway via glucose 6-phosphate dehydrogenase [58,82]. Reduced form of trypanothione T(SH)_2_ is used by the tryparedoxin/tryparedoxin peroxidase system (TXN/TXNPx) to reduce hydrogen peroxide, alkyl-hydroperoxide and other reactive oxygen species produced by the macrophage. The T(SH)_2_/TryR/TXN/TXNPx system replaces many of the antioxidant and metabolic functions of glutathione GSH/glutathione reductase GR and thioredoxin/thioredoxin reductase systems present in mammals (Figure 6) [80,81,83].

It has been shown that TryR, TryS and TXN/TXNPx are essential for the survival of parasites by protecting them against oxidative stress [58]. The enzymes of the trypanothione pathway are absent in humans but essential for parasite survival, therefore they are considered among the best targets for the discovery of antileishmanial drugs [80]. The enzyme trypanothione reductase TryR was described in 1985 [84] and is a validated drug target in trypanosomatid diseases [73]. Thus, in order to investigate the mechanism of action, the mesoionic derivatives **37**, **38**, **42** and **45** studied before were evaluated to determine their effect on TryR activity. Three species of *Leishmania* were selected: *L. amazonensis*, *L. braziliensis*, and *L. infantum*. Among the studied compounds, only the nitro-derivative **45** exhibited enzyme inhibition in extracts from cultures of parasites. At a concentration of 1 μM, the nitro-derivative **45** was able to inhibit 76% of NADPH consumption by TryR in *L. amazonensis* extracts, 70% in *L. infantum* and 69.5% in *L. braziliensis* compared with the control. The enzyme kinetics determined with *Li*TryR demonstrated a non-competitive inhibition profile with IC_50_ value of 1.63 μM. These results indicate that TryR could be a molecular target for the derivative **45**. Molecular docking studies have indicated that these mesoionic compounds could effectively fit into the substrate binding site together with the substrate molecule [73]. The derivative **45** was also active on *L. amazonensis* promastigotes with IC_50_ value of 1 μM [67].

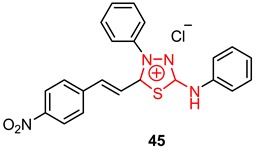



However, TryR has structural similarity to human glutathione reductase GR, which can make it difficult to design selective derivatives against this enzyme [58]. Under these conditions, TryS remains the most promising target since it is a low-abundance, essential enzyme in *Leishmania* with no human homologs [83]. The importance of TryS activity for the viability of the parasites has been demonstrated in vitro and in vivo by genetic and pharmacological methods on *T. brucei brucei* and *L. infantum* [82]. A kinetic model of trypanothione T(SH)_2_ metabolism in *T. cruzi* predicted that in order to diminish the T(SH)_2_ synthesis by 50%, it is necessary either to inhibit TryS by 63% or more than 98% of TryR. It seems that moderate inhibition of TryS might be a promising target in drug development. Instead, highly potent and specific TryR inhibitors are needed to affect the antioxidant capabilities of the parasites [85]. In addition, TryS has several advantages as a molecular target candidate in drug development: it is encoded by a single copy gene, the TryS structure of *L. major* has been elucidated, TryS has been shown to provide metabolic control to the trypanothione pathway in *T. cruzi* and kinetic information is available for multiple TryS [82,85,86,87,88].

#### 3.4.3. Pteridine Reductase-1 Inhibitors

Scientific works in the field of antiparasitic therapy has demonstrated that inhibition of enzymes involved in the parasites life cycle, such as dihydrofolate reductase (DHFR), thymidylate synthase (TS) and pteridine reductase (PTR1) should provide effective treatment. Even if DHFR inhibitors (antifolates) can severely affect DNA replication resulting in cell death, they are not currently used in the antiparasitic therapy mainly because of the PTR1 activity of the target organisms. PTR1 is a dehydrogenase/reductase that is able of effecting successive reductions of both conjugated (folate) and unconjugated (biopterin) pterins [68]. *Leishmania* protozoan parasites are auxotrophic for both folates and unconjugated pteridines. *Leishmania* saves these metabolites from their mammalian hosts and insect vectors through several transporters. Within the parasite, folates are reduced by a bifunctional DHFR-TS enzyme and by PTR1 [89]. On the other hand, biopterin taken from the host is sequentially reduced to dihydrobiopterin (H_2_B) and tetrahydrobiopterin (H_4_B) by PTR1 [90]. Genetic studies on *L. major* have demonstrated that PTR1 is essential for the growth of parasitic promastigotes. In the case of *Leishmania* mutants lacking the PTR1 enzyme, the growth of promastigotes in culture is not possible unless supplementary of H_2_B or H_4_B is added. These mutants gave more infectious parasites that produced larger lesions when injected into mice. This indicates that *Leishmania* mutants lacking the PTR1 enzyme are viable *in vivo*, probably due to abundant H_4_B in host macrophages and consequently that PTR1 alone is not a drug target in *L. major* [90,91].

Previous studies have shown that simultaneous inhibition of parasitic DHFR and TS ensures the parasites survival through PTR1 overexpression [92,93]. The amplification of PTR1 in *Leishmania* spp. is one of several mechanisms by which parasites acquire resistance to antifolate drugs [90]. Since PTR1 can act on a broader range of substrates, this suggests that simultaneous inhibition of parasitic DHFR and PTR1 could result in the death of parasites [92,93]. Ferrari et al. [68] made a virtual screening combined with experimental studies to identify *L. major* PTR1 (*Lm*PTR1) inhibitors as potential antiparasitic agents. A virtual calculated ternary complex of *Lm*PTR1-NADPH-2-amino-1,3,4-thiadiazole showed the ligand to be involved in multiple interactions. Based on this model, the 5 position of 2-amino-1,3,4-thiadiazole scaffold has huge potency to bind different types of substituents. Taking into account the inhibitory activity of 2-amino-1,3,4-thiadiazole **49** against *Lm*PTR1 (IC_50_ value of 5600 μM), different 2-amino-5-R-1,3,4-thiadiazole derivatives were synthesized. The compounds were tested against *L. major* enzymes—*Lm*PTR1 and *Lm*DHFR, and against the human DHFR (hDHFR) [68].

The derivatives **49**–**54** showed competitive inhibition of *Lm*PTR1 with IC_50_ values between 22 and 309 μM (IC_50_ values: **50**—31 μM; **51**—309 μM; **52**—22 μM; **53**—29 μM; **54**—89 μM). All compounds were more active compared to 2-amino-1,3,4-thiadiazole **46**. The screening of *Lm*DHFR inhibition revealed that these derivatives have low or no specificity for *Lm*DHFR. Only two derivatives, **52** and **53** inhibited *Lm*DHFR with IC_50_ values of 1300 μM and 139 μM, respectively. Fortunately only one derivative, **53**, exhibited an inhibitory activity against hDHFR (IC_50_ value of 300 μM). From these results it can be concluded that this class of derivatives are selective *Lm*PTR1 inhibitors [68].

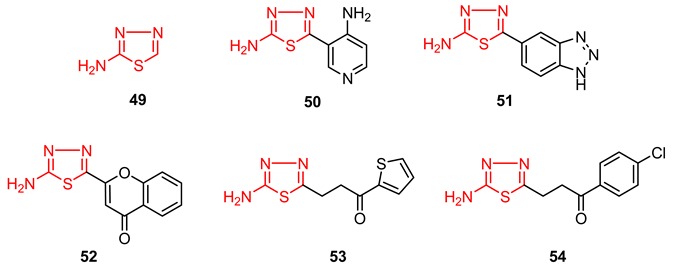



The selected compounds **50**–**54**, both as single agents and in combination with pyrimethamine—5-(4-chlorophenyl)-6-ethyl pyrimidine-2,4-diamine, were also studied for their inhibitory activity against the promastigotes of *L. mexicana* and *L. major*. As single agents, the compounds were inactive or moderately active on parasites growth (e.g., derivatives **51**, **52** and **53** exhibited a moderate effect on *L. major* promastigotes with an inhibition rate of 14.9% to 24.0% at a concentration of 50 μg/mL). Pyrimethamine is a known DHFR inhibitor used as antimalarial drug and with no activity in leishmaniasis. However, the combination of 1,3,4-thiadiazole derivatives **50**–**54** with pyrimethamine resulted in the growth inhibition of both *L. mexicana* (growth inhibition in the range 65–85%) and *L. major* (growth inhibition in the range 59–85%) promastigotes. The best results were obtained for the combination of 30 μg/mL of pyrimethamine with 50 μg/mL of compound **50** (85% inhibition of growth of *L. mexicana* promastigotes) and 30 μg/mL of pyrimethamine with 50 μg/mL of compound **52** (85% inhibition of growth of *L. major* promastigotes). In addition, compound **50** exhibited a very low toxicity against the human fibroblasts MRC-5 (10% inhibition of growth of MRC-5 cells at 100 μg/mL), suggesting a better safety profile compared to other studied compounds. These results show that selective *Lm*PTR1 inhibitors alone do not exhibit antiparasitic activity and suggest that the combination of DHFR inhibitors with PTR1 inhibitors might be an effective treatment for leishmaniasis and possibly other parasitic diseases [68].

## 4. Conclusions

Leishmaniasis is a serious public health problem in developing countries of tropical and subtropical areas and in economically disadvantaged regions [46,59]. Tourism, business travel and immigration have led to the spreading of leishmaniasis in the industrialized world among populations that are not immune to these parasites [59]. Although significant advances have been made in the treatment of leishmaniasis over the last decade, the available antileishmanial drugs require long-term treatment, high cost and cause serious side effects due to toxicity [70]. The antileishmaniasis therapy remains a major unmet medical need, particularly for people with a weak immune system and lack of financial resources. Early diagnosis and access to safe medicines increase the chances of treatment and survival of affected people [59]. The lack of an authorized vaccine, together with a lack of effective medication and a significant drug resistance worldwide, has made it imperative to research for new, inexpensive, efficient and safe antileishmanial drugs [47,60]. Thiadiazole is a versatile moiety and the thiadiazole derivatives have been widely studied for medical, agricultural and industrial applications. Literature surveys report the antileishmanial properties of 2-amino-1,3,4-thiadiazole derivatives and SAR studies have shown the structural units that are important for pharmacological activity. In addition, a good solution to avoid drug resistance could be the development of new drugs with new mechanisms of action. A new strategy that could be used to combat leishmanial infections is the targeting of enzymes that are essential for the survival of parasites and several candidates with 2-amino-1,3,4-thiadiazole skeleton are identified as parasitic enzymes inhibitors. Although further studies are needed to confirm the quality of 2-amino-1,3,4-thiadiazole derivatives as a new class of antileishmanial agents, these results open new ways for the development of therapeutic approaches to manage leishmanial infections by novel technologies.

## Figures and Tables

**Table 1:** Structural details, IC_50_ values and CC_50_ values of compounds **4**–**25**.

	R	IC_50_ (μM)	CC_50_ (μM)		R	IC_50_ (μM)	CC_50_ (μM)
**4**	Me	54 ± 0.17	-	**16**	Me	64 ± 0.32	-
**5**	Et	50 ± 0.8	-	**17**	Et	98.4 ± 0.12	-
**6**	*c*-Pr	55.5 ± 0.66	-	**18**	*c*-Pr	97.3 ± 0.98	-
**7**	(CH_2_)_2_OH	21 ± 0.65	122.11	**19**	(CH_2_)_3_OH	3 ± 0.41	42.16
**8**	(CH_2_)_3_OH	18 ± 0.2	62.33	**20**	(CH_2_)_3_OMe	3 ± 0.5	37.80
**9**	CH_2_CH(OMe)_2_	13 ± 0.53	62.46	**21**	CH_2_CH(OMe)_2_	56 ± 0.74	-
**10**	(CH_2_)_2_N(Et)_2_	44 ± 0.72	83.29	**22**	(CH_2_)_2_N(Et)_2_	36 ± 0.24	43.80
**11**	(CH_2_)_3_N(Et)_2_	13 ± 0.43	104.60	**23**	(CH_2_)_3_N(Me)_2_	25 ± 0.17	87.35
**12**	(CH_2_)_2_N(*i*-Pr)_2_	13 ± 0.23	42.18	**24**	(CH_2_)_2_O(CH_2_)_2_OH	42 ± 0.45	-
**13**	CH(Et)CH_2_OH	10 ± 0.66	83.29	**25**	CH_2_CH=CH_2_	34.3 ± 0.5	129.32
**14**	(CH_2_)_2_O(CH_2_)_2_OH	116 ± 0.76	-				
**15**	CH(Me)(CH_2_)_3_N(Et)_2_	33 ± 0.76	367.65				

**Table 2:** Details and IC_50_ values of compounds **26**–**34**.

	R	IC_50_ (μM)
**26**	H	19.6 ± 0.56
**27**	2-F	19.0 ± 0.35
**28**	4-F	21.4 ± 1.86
**29**	2-Cl	18.9 ± 0.12
**30**	4-Cl	88.8 ± 0.9
**31**	2-Me	25.2 ± 0.54
**32**	4-Me	12.2 ± 0.66
**33**	2,4-Cl_2_	19.9 ± 0.84
**34**	2-F-6-Cl	17.4 ± 0.76

**Table 3:** Details, IC_50_ values, ED_50_ values and TD_50_ values of compounds **37**–**45** [61,67].

	X	Y	*L. amazonensis*		*L. braziliensis* ED_50_ (μM)	*L. chagasi* ED_50_ (μM)	TD_50_ (μM)
			Promastigotes IC_50_ (μM)	Amastigotes IC_50_ (μM)			
**37**	H	H	0.47 ± 0.03	104.54 ± 11.95	49.61	22.76	3.06
**38**	OMe	H	0.17 ± 0.01	23.93 ± 4.88	46.20	8.31	1.13
**39**	F	H	0.92 ± 0.06	5.37 ± 0.28	5.1	3.42	5.82
**40**	Cl	H	1.51 ± 0.22	186.34 ± 18.11	6.23	13.17	11.98
**41**	Br	H	0.87 ± 0.1	33.26 ± 2.38	2.93	9.93	6.59
**42**	H	OMe	0.04 ± 0.01	41.88 ± 2.83	30.64	4.75	0.61
**43**	H	Cl	0.48 ± 0.05	178.11 ± 15.39	1.72	5.17	6.23
**44**	H	Br	0.52 ± 0.05	5.48 ± 0.04	25.95	144.68	2.47
**45**	NO_2_	H	1.00 ± 0.12	52.92 ± 5.92	70.77	22.83	5.49
Pentamidine			0.46 ± 0.08	118.00 ± 7.31	-	-	-
Pentamidine (Filaxis Lab)			23.64/ED_50_	-	64.18	27.5	2.21
